# Regulation of INPP5E in Ciliogenesis, Development, and Disease

**DOI:** 10.7150/ijbs.99010

**Published:** 2025-01-01

**Authors:** Abdulaziz Hakeem, Shuying Yang

**Affiliations:** 1 Department of Basic & Translational Sciences, School of Dental Medicine, University of Pennsylvania, USA.; 2 Department of Basic and Translation Science, School of Dentistry, Umm Al Qura University, Saudi Arabia.; 3 The Penn Center for Musculoskeletal Disorders, Perelman School of Medicine, University of Pennsylvania, Philadelphia, USA.; 4 Center for Innovation & Precision Dentistry, Penn Dental Medicine and School of Engineering and Applied Sciences, University of Pennsylvania, Philadelphia, USA.

**Keywords:** *INPP5E*, inositol polyphosphate-5-phosphatase E, disease, cell behavior, primary cilia

## Abstract

Inositol polyphosphate-5-phosphatase E (INPP5E) is a 5-phosphatase critically involved in diverse physiological processes, including embryonic development, neurological function, immune regulation, hemopoietic cell dynamics, and macrophage proliferation, differentiation, and phagocytosis. Mutations in *INPP5E* cause Joubert and Meckel-Gruber syndromes in humans; these are characterized by brain malformations, microphthalmia, situs inversus, skeletal abnormalities, and polydactyly. Recent studies have demonstrated the key role of INPP5E in governing intracellular processes like endocytosis, exocytosis, vesicular trafficking, and membrane dynamics. Moreover, it regulates cellular signaling pathways by dephosphorylating the 5-phosphate of phosphatidylinositol-3,4,5-trisphosphate, phosphatidylinositol 4,5-bisphosphate, and phosphatidylinositol 3,5-bisphosphate. Despite recent advances, knowledge gaps persist regarding the function and molecular mechanism of INPP5E in various cells and species. This review integrates recent findings on the role of INPP5E in regulating cellular function, development, and the pathogenesis of various human disorders, emphasizing the molecular mechanism by which INPP5E regulates primary cilia assembly and function and critical signaling pathways. Identifying the importance of INPP5E in healthy and diseased states can advance our understanding of cellular processes and disease pathogenesis and provide a foundation for developing targeted therapeutic interventions.

## 1. Introduction

Inositol polyphosphate 5-phosphatases (INPP5s) represent a superfamily of phosphatases that remove the fifth-position phosphate from phosphatidylinositol-3,4,5-trisphosphate [PI(3,4,5)P3], phosphatidylinositol 4,5-bisphosphate [PI(4,5)P2], and phosphatidylinositol 3,5-bisphosphate [PI(3,5)P2] to form phosphatidylinositol 3,4-bisphosphate [PI(3,4)P2], phosphatidylinositol 4-phosphate [PI(4)P], and phosphatidylinositol 3-phosphate [PI(3)P], respectively 1. INPP5s are involved in multiple cellular processes, including ion channel control, membrane trafficking, synaptic vesicle formation, hematopoietic cell proliferation, and signal transmission 2 by regulating inositol polyphosphate and phosphatidylinositol signaling 3, 4. The *INPP5* family includes *INPP5A* (also called proline-rich *INPP5* or *PIPP*), *INPP5B*, *INPP5C*, *INPP5D* (also known as SH-2 containing inositol 5′ polyphosphatase 1 or SHIP1), *INPP5E* (also called PIPP1), *INPP5F* (also called SAC2), *INPP5J* (also referred to as PIPP2), and *INPP5K* (SKIP) members 5. These proteins have non-redundant roles in regulating cell functions and important signaling pathways. The crucial roles of these enzymes in biological processes have been summarized elsewhere 6-11.

Phosphatidylinositol-4,5-bisphosphate 5-phosphatase E (INPP5E), also known as pharbin, is a 72-kDa protein comprising 644 amino acids in humans. It is encoded by *INPP5E* on chromosome 9. *INPP5E* was first identified by Kisseleva *et al.* in 2000 10. Subsequently, increasing evidence has shown that INPP5E regulates various cellular activities, including ion channel function, membrane trafficking, synaptic vesicle formation, hematopoietic cell proliferation, and signal transmission 10, 11. INPP5E mutations are associated with various pathologies, including Joubert syndrome, human syndromic ciliopathies, mental retardation, truncal obesity, retinal dystrophy, and micropenis (MORM) syndrome 11-14. These findings highlight the importance of the spatial localization and enzymatic activity of INPP5E within cilia 15, 16. However, gaps remain regarding how INPP5E functions in healthy cells and how its inactivation leads to diseases. In this review, we comprehensively summarize the role of INPP5E in regulating cellular proliferation, differentiation, and function and highlight the importance of INPP5E in ciliogenesis. We also discuss the contribution of INPP5E to organ development and disease pathogenesis, including ciliopathies, Joubert syndrome, polycystic kidney disease (PKD), sonic hedgehog (Shh) medulloblastoma, cystic renal dysplasia, hepatic fibrosis, colorectal carcinoma, inherited retinal degeneration (IRD), MORM syndrome, and inflammation. Finally, we discuss the signaling pathways regulated by INPP5E under physiological and pathophysiological conditions.

## 2. Methods

A literature search was performed using “PubMed,” “Embase,” and “Scopus” with the following search terms: “Inositol Polyphosphate-5-Phosphatase E” and “INPP5E gene.” The search yielded 360 articles. The inclusion criteria included studies published up to 2024 that contained specific keywords, such as “INPP5E” and “inositol polyphosphate-5-phosphatase E.” Additionally, only studies published in English and those specifically focused on INPP5E were considered. Studies that mentioned INPP5E but did not study or investigate it directly were excluded. After review, 49 articles describing specific studies on INPP5E were included. The other articles served as sources of background information.

## 3. INPP5E expression in organelles and tissues

**Figure 1:**
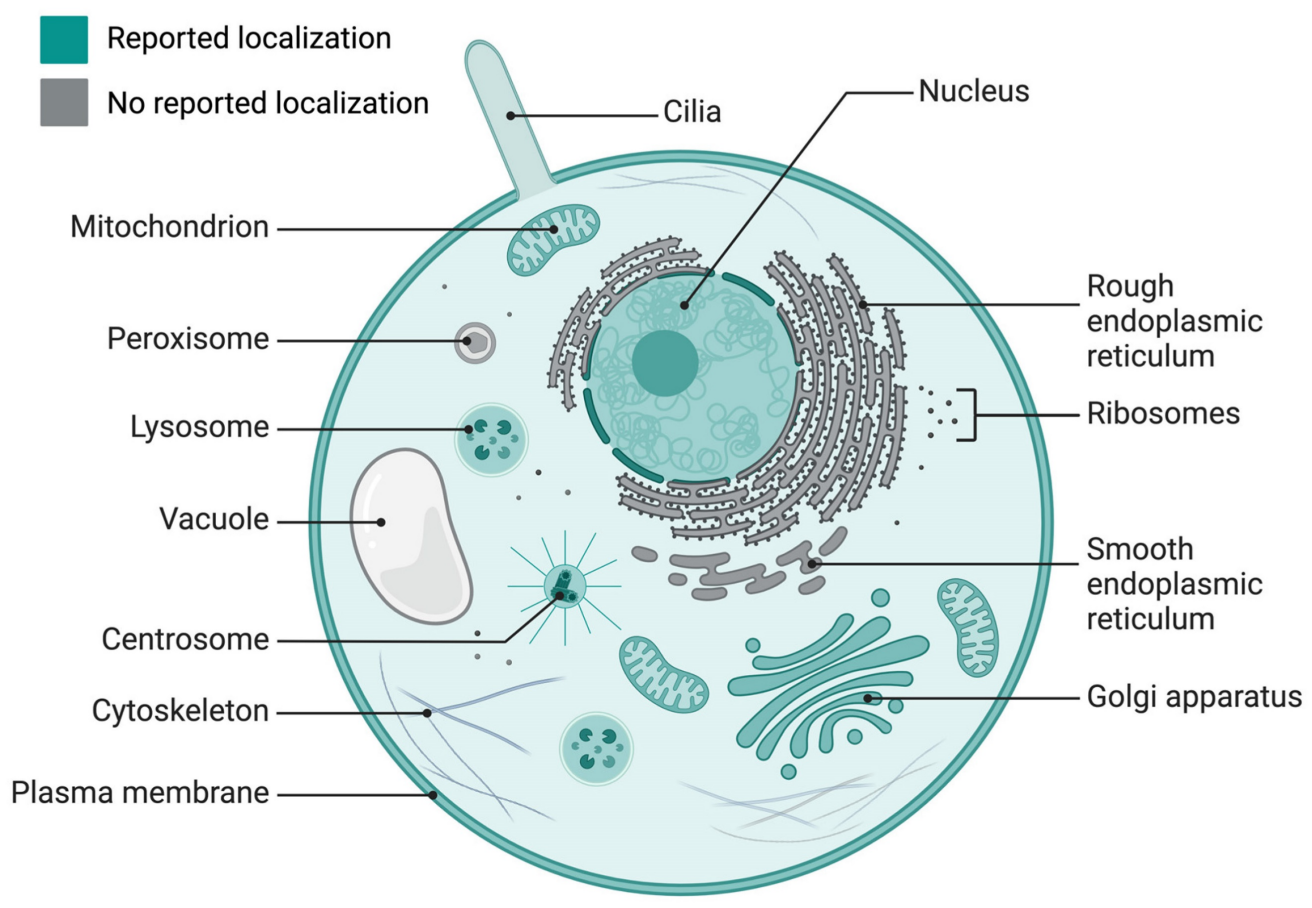
** INPP5E localization in the cell.** The literature-reported localization of INPP5E in a cell is presented in green (cilia, Golgi apparatus, mitochondria, lysosome, centrosome, nucleus, and plasma membrane); the unreported localization appears in gray/white (rough and smooth endoplasmic reticulum, ribosome, cytoskeleton, and peroxisome). *Created in BioRender. Hakeem, A. (2024) BioRender.com/h58k985*.

INPP5E expression has been reported in different cellular organelles, including the Golgi apparatus 17, 18, lysosomes 19, 20, membranes 21, 22, nucleus 23, and centrosomes 23 (Fig. 1). Additionally, INPP5E localizes in many cells and tissues, such as epithelial cells 4, 22, 24-26, Kupffer's vesicles, pronephric ducts 4, retinal and renal tissue 24, kidneys 22, pronephric ducts, otic vesicles 25, colorectum 26, and neuroepithelium in the cerebellum 27. Furthermore, it is expressed in immune cells, including macrophages 21 and T-cells 28, in addition to photoreceptor cells in the inner segment tissue of the retina 29, mesenchymal cells in the hindlimbs and ribcage 30, and hepatocytes in the liver 31. *INPP5E* is also reportedly expressed in different neural cells 24, 30, 32-35.

INPP5E protein is found in different locations within cilia. In mouse embryonic fibroblasts, INPP5E has been reported to partially localizes to the transition zone (TZ) near specific lipid molecules (PI(4,5)P2 and PI(3,4,5)P3) during Hedgehog (Hh) pathway activation 30. INPP5E is localized to the apical membrane and basal bodies, not cilia, in the pronephric epithelium of zebrafish to break down PI(3,4,5)P3 25. INPP5E is also present in the ciliary membrane, directly interacting with ARL13B 36. In IMCD3 cells, INPP5E primarily localizes to the ciliary axoneme and, to a lesser extent, the basal end of cilia 37, mediated by the intraflagellar transport (IFT) system 38.

## 4. Regulation of INPP5E during ciliogenesis

### 4.1. Structure and function of primary cilia

**Figure 2:**
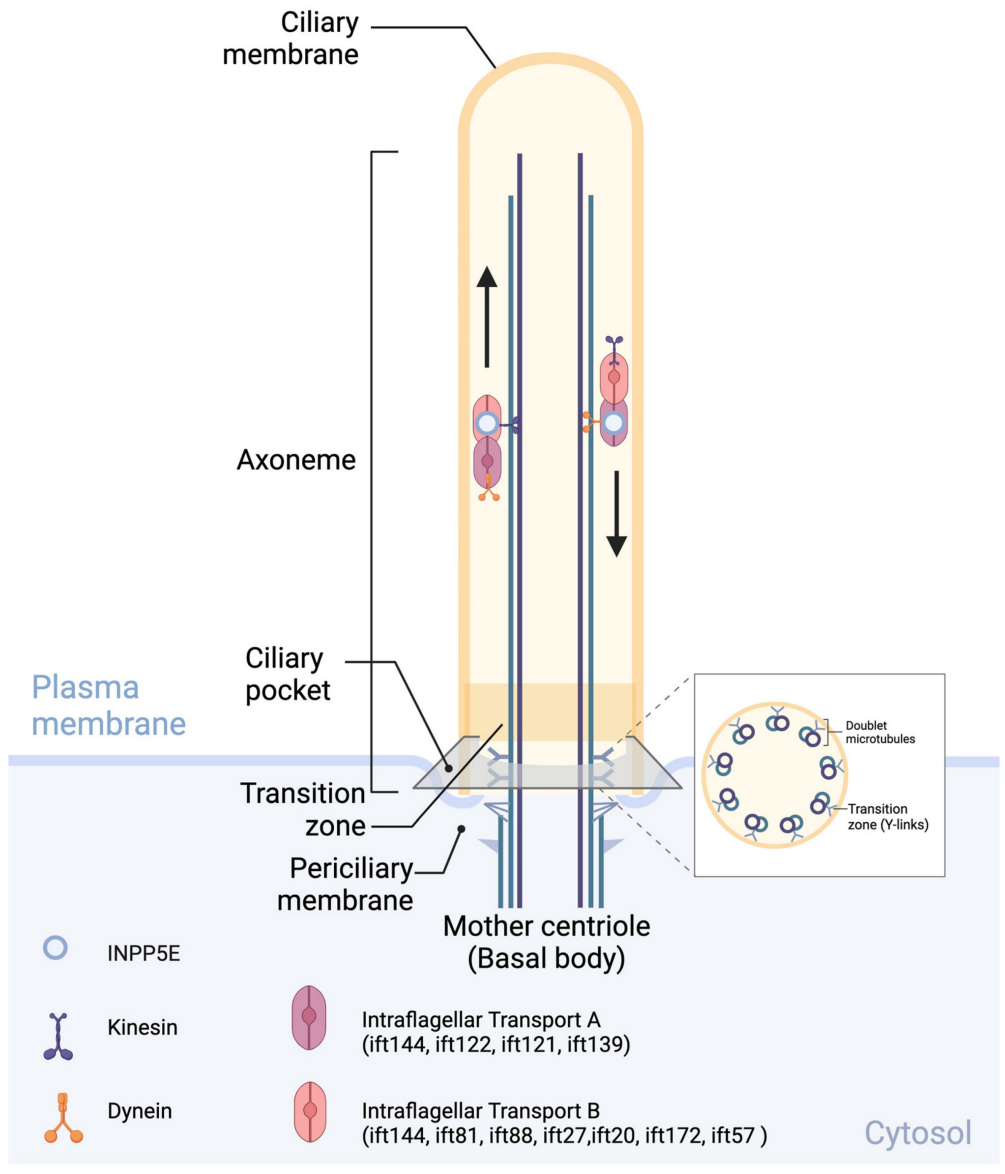
** Primary cilia structure:** Intraflagellar transport B (IFT-B), such as IFT144, IFT81, IFT88, IFT27, IFT20, IFT172, and IFT57, as well as Intraflagellar transport A (IFT-A), including IFT144, IFT122, IFT121, and IFT13, contribute to INPP5E transport; their components and machinery are essential for the assembly, maintenance, and function of primary cilia. *Created in BioRender. Hakeem, A. (2024) BioRender.com/d83l169*.

The primary cilium is a dynamic and sensory subcellular organelle that extends from the cell membrane surface (Fig. 2) 35, 39. Primary cilia comprise four essential components 39 including basal body, axoneme, transition zone (TZ) and ciliary membrane. The basal body develops from the mother centriole and serves as an anchor for the ciliary membrane. The axoneme consists of a ring of nine outer microtubule doublets and provides a scaffold for multiple protein complexes such as Intraflagellar Transport B (IFT-B) and Intraflagellar Transport A (IFT-A), crucial components of the IFT machinery and their associated motor proteins dynein and kinesin. The IFT-B complex, including ift144, ift81, ift88, ift27, ift20, ift172, and ift57, moves cargo from the base of the cilium to the tip using kinesin motor proteins, whereas the IFT-A complex, including ift144, ift122, ift121, and ift139, moves cargo from the tip of the cilium back to the base using dynein motor proteins. The ciliary sheath covers axoneme and it is a continuous with the plasma membrane and the TZ (the region between the basal body and axoneme) separate them functionally 29, 39. The TZ in primary cilia, also known as the ciliary gate, is a critical region located at the base of the cilium and is characterized by Y-shaped linkers, a ciliary necklace, and transition fibers. This region functions as a selective gate that controls transport, acts as a diffusion barrier, and regulates signaling pathways within the cilium 39. Finally, the ciliary membrane covers the microtubule-based axoneme 24-29.

Primary cilia are essential for vertebrate embryonic development as they coordinate multiple signaling cascades 35. Primary cilia dynamics are closely linked to cell cycle progression, assembling and disassembling when cells exit and enter the cell cycle, respectively 39. In non-dividing cells, primary cilia maintain the balance of phosphoinositide, which serves as a second messenger in signal transduction 24, 25. As a cellular antenna and environmental sensor, primary cilia can sense changes in external mechanical (physical) stimuli such as mucus, fluid flow, and chemical signals (such as hormones and nutrients) and transmit the signals between cells, which can lead to cellular responses 39. The sensory function of primary cilia is essential for embryonic development, sensory perception, and tissue balance. Moreover, primary cilia help regulate the balance of various organs and tissues in vertebrates 29, 35.

**Figure 3:**
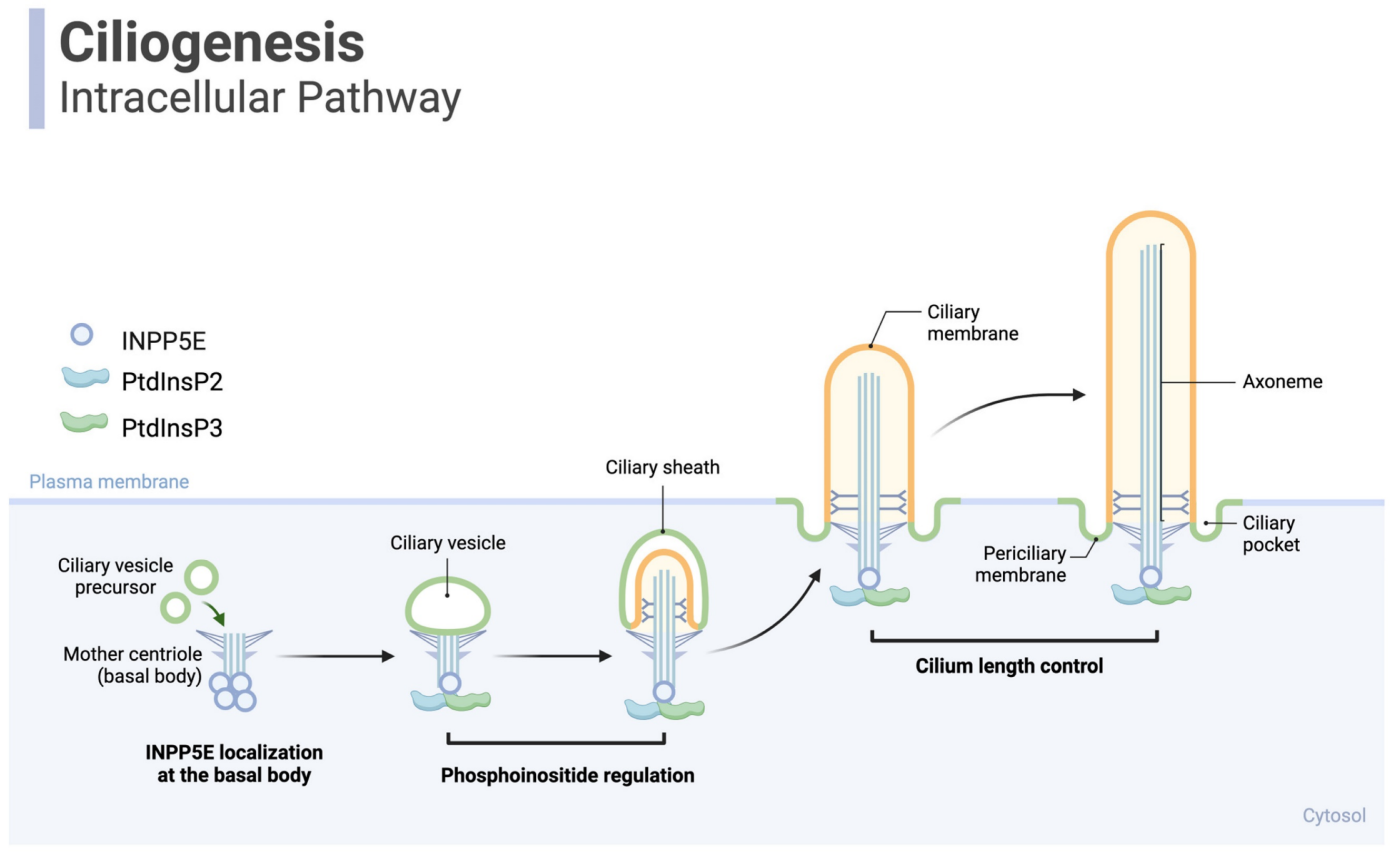
** Ciliogenesis: INPP5E is a crucial protein in developing primary cilia.** Primary cilia are microtubule-based organelles extending from the surfaces of most mammalian cells and are involved in various cellular signaling pathways. Inositol polyphosphate-5-phosphatase E (INPP5E) is localized to the base of primary cilia, acting as a phosphoinositide phosphatase to explicitly target the lipid molecules phosphatidylinositol 4,5-bisphosphate (PIP2) and phosphatidylinositol 3,4,5-bisphosphate (PIP3). It dephosphorylates PIP2 and PIP3, converting them to PI4P. Proper levels of PIP2 are crucial for localizing various proteins involved in cilia assembly, including intraflagellar transport (IFT) machinery, which facilitates the trafficking of components required for cilium elongation and maintenance. INPP5E also helps control cilium elongation. Regulating PIP2 levels influences the activation of various downstream signaling pathways that control protein trafficking into and out of the cilium. This regulation of protein trafficking contributes to the modulation of cilium length. *Created in BioRender. Hakeem, A. (2024) BioRender.com/g24c237*.

INPP5E is critically involved in multiple cellular processes, particularly ciliogenesis and the microtubule assembly of tactile organelles in most mammalian cells (Fig. 3) 35. INPP5E is essential for cilia formation and function across various tissues in myriad physiological processes, including regulating lipid levels 39. INPP5E controls the lipid and protein connections at the ciliary base and participates in hydrolyzing two key phospholipids—PI(3,4,5)P3 and PI(4,5)P2 into PI(3,4)P2 and PI(4)P, respectively 39. These products are vital for preserving the structure and length of primary cilia, especially in dormant cells 39.

### 4.2. Role of INPP5E in ciliogenesis in different animal models

#### 4.2.1. Inpp5e regulates ciliogenesis during zebrafish development

Roles for *inpp5e* in zebrafish ciliogenesis have been defined. Luo *et al.* 4 first revealed that *inpp5e* is required for ciliogenesis in zebrafish kidneys. They found that suppressing *inpp5e* expression with morpholinos results in shorter and fewer cilia in the Kupffer's vesicle of young larvae and the pronephric ducts of the kidneys, as well as impaired epinephrine-induced melanosome retraction 4. Zebrafish with an *inpp5e* mutation exhibit abnormal ocular size, kidney function, heart development, eye development, and body symmetry 4. Additionally, Xu *et al.* 25 reported that zebrafish *inpp5e* promotes ciliogenesis in renal epithelium by breaking down PI(3,4,5)P3 and stabilizing apical PI(4,5)P2, helping recruit Ezrin, F-actin, and basal bodies to the apical side of the renal epithelium. Meanwhile, downregulating *inpp5e* in zebrafish causes abnormalities in cilia formation and leads to cystic kidney development 25. Moreover, *inpp5e* is crucial in suppressing PI(3,4,5)P3 expression in the apical membrane of the pronephric epithelia in zebrafish embryos 25. When *inpp5e* is absent or depleted, PI(3,4,5)P3 is localized at the apical membrane, causing PI3K to relocate via a positive feedback mechanism to convert PI(4,5)P2 to PI(3,4,5)P3, decreasing PI(4,5)P2 levels 25. These findings provide evidence that *inpp5e* is essential for ciliogenesis and organ development and function in zebrafish.

#### 4.2.2. Inpp5e regulates ciliogenesis during mouse development

Consistent with the findings in zebrafish, *Inpp5e* is also required for ciliogenesis in mouse organs (i.e., brain and retina) and cells (i.e., inner medullary collecting duct cells, NIH3T3 cells, fibroblasts, and rod outer segments) 21-25. Xu *et al.* 24 conditionally deleted *INPP5E* from human renal cortical tubular epithelial cells and 293 cells, causing shortened cilia and ciliary disassembly in inner medullary collecting duct cells. They showed that phosphatidylinositol-4-phosphate 5-kinase type I (PIPKI) and INPP5E regulate PI(4)P lipid levels in centrosomes, which develop into primary cilia 24. PIPKI and INPP5E also localize tau tubulin kinase 2 (TTBK2) to the cilial basal body 24. Furthermore, for functional cilia to develop, centriolar coiled-coil protein 110, which blocks axoneme formation in cilia, must be absent, and TTBK2 must be present 24. The ciliary assembly protein centrosomal protein 164 (CEP164) binds to PI(4)P, preventing its interaction with TTBK2 24. Therefore, PIPKI and INPP5E regulate PI(4)P-dependent TTBK2 recruitment to initiate ciliogenesis, and INPP5E improves the coordination of ciliogenesis in mouse inner medullary collecting duct and renal cortical tubular epithelial cells 24.

Similar findings have shown that INPP5E is necessary for ciliogenesis during mouse embryonic neurodevelopment 35. Organoids harboring an *Inpp5e* mutation exhibit fewer cilia and extra ciliary smoothened (SMO) proteins 40. This alters the expression of several cilium-related genes, including *Kras*, *Ift80*, *Pkhd1*, *Prkca*, *Mkks*, and *Smo* 41. Most importantly, radial glial cells exhibit abnormal sonic hedgehog (*Shh*) signaling 35. During neural tube closure, INPP5E expression is markedly decreased in mice with abnormal neurodevelopment and/or neural tube defects compared to normal control mice 35. This finding suggests that decreased expression of *Inpp5e* is closely associated with neural tube deformities, and INPP5E is essential for ciliogenesis and brain development 35.

INPP5E is also strongly associated with the development and function of other organs. Sharif *et al.* 29 recently reported that *Inpp5e* deletion in the retina results in a regular-length connecting cilium; however, this does not extend axonemes into the outer segment (OS) or form disks 29. Moreover, the deletion of *Inpp5e* in neural stem cells impairs cilia formation and results in the accumulation of PI(4,5)P2, tubby-like protein 3 (TULP3), and IFT particles, inhibiting OS growth 29. Furthermore, Dewees *et al.* 18 reported that the transportation of IFT140 and INPP5E to the cilia requires ADP-ribosylation factor-like GTPase (ARL) 16, whereas INPP5E relies solely on retinal rod rhodopsin-sensitive cGMP 3′,5′-cyclic phosphodiesterase subunit delta (PDE6D). Consequently, the prenylated INPP5E cargo binds to the PDE6D shuttle 18. The removal of *Arl16* from mouse embryonic fibroblasts (MEFs) inhibits ciliogenesis and alters ciliary protein content, resulting in a loss of ARL13B, ARL3, INPP5E, and the IFT-A core component IFT140 18. Hence, INPP5E is critical in organ development, regulating cilia formation and function 18.

## 5. Role of INPP5E in cellular function

INPP5E plays crucial roles in regulating cellular functions, such as inflammation, immune cell responses, phagocytosis, autophagy, the functionality of olfactory sensory neurons, and the cell cycle, highlighting its importance in maintaining cellular homeostasis.

### 5.1. Role of INPP5E in inflammation and immune cells

The inositol family has well-defined effects on immune cell function. Specifically, the role of inositol 1,4,5-triphosphate hydrolysis and Ca^2+^ release in immune cells, specifically macrophages, was first described in 1986 by Kukita *et al.* 42. The study examined the role of Ca^2+^ in the hydrolysis of PI(4,5)P2 in isolated guinea pig macrophages stimulated with fMet-Leu-Phe. This potent chemotactic factor attracts immune cells, such as macrophages, to infection or inflammation sites and participates in degranulation, superoxide production, and intracellular calcium mobilization. fMet-Leu-Phe causes a rapid decrease in PIP2 levels, accompanied by phosphatidic acid and IP3 accumulation 42.

INPP5E is crucial for the formation and function of immune synapses, specialized interfaces between T lymphocytes and antigen-presenting cells 28. INPP5E is enriched in cilia and regulates the localization of phosphoinositides 28. It is highly concentrated at the immune synapse in Jurkat T-cells in antibody-mediated crosslinking of T-cell receptor (TCR) complexes or superantigen-mediated conjugation 28 and regulates immune synapses by interacting with CD3ζ, ZAP-70, and Lck. Repressing INPP5E in T-cells disrupts CD3ζ polarized distribution at the immune synapse and causes failed PI(4,5)P2 clearance at the center of the synapse 28. Additionally, silencing INPP5E reduces proximal TCR signaling and decreases interleukin (IL)-2 secretion 28. These findings highlight the significant role of INPP5E in manipulating phosphoinositides at the synapse, especially in controlling the TCR signaling cascade.

INPP5E regulates hematopoietic lineage by regulating centrosome interactions with immune synapses in lymphocytes 28, 43. Chiu *et al.* reported that INPP5E is crucial in T-cell activation, regulating phosphoinositide distribution at the immune synapse 28. This helps in the polarized distribution of CD3ζ, a vital component of the TCR complex. Moreover, INPP5E actively participates in the formation of CD3ζ, ZAP-70, and Lck complexes at the immune synapse to properly activate the TCR signaling cascade. Given its role as a primary activating signal for T-cells, CD3ζ is used as the driver of signal transmission by all currently approved chimeric antigen receptor T-cells targeting CD19 or B-cell maturation antigens. These findings highlight the crucial role of the ciliary-enriched phosphatase INPP5E in immune synapses and suggest that chimeric antigen receptor T-cell therapy in relation to INPP5E is a promising strategy for immunotherapy 28, 43.

INPP5E also regulates viral infections and their mediated immune responses. Several viruses trigger PI3K signaling pathways to elevate PI(3,4,5)P3 levels, activating the protein kinase B (AKT)-mammalian target of rapamycin (mTOR) signaling axis and impacting infection efficiency 44. PI(4,5)P2 and PI(3,4,5)P3 are INPP5E substrates and well-defined mediators of actin remodeling 44. Hoang *et al.* revealed that the removal of INPP5E enhances herpes simplex virus type 1 and vesicular stomatitis virus D51 infection in 4T1 cells 44 by altering the actin cytoskeleton and membrane ruffling, improving viral attachment and enhancing binding of the viruses to the cell surface 44. Despite INPP5E's localization to the cilium, viral attachment does not show separation as expected if enhanced attachment occurs at or close to ciliary structures. Thus, INPP5E's antiviral outcomes may not be connected to its ciliary localization. Centrosomal PIs can also be influenced by INPP5E loss, causing spindle microtubule deterioration, potentially through a discrepancy in PI(4,5)P2 expression. Whether these immune mechanisms mediate enhanced virus binding and infection is subject to future investigation.

### 5.2. Role of INPP5E in phagosome function

Studies have shown that INPP5E influences macrophage phagocytosis and T-cell synapse by regulating the TCR signaling cascade 21, 28. Decreased INPP5E levels (shInpp5e cells) can result in reduced RAS-related protein RAB20 levels in phagosomes, accelerating phagosome acidification 21. Moreover, PI(3,4,5)P3 and PI(3,4)P2 levels become altered during phagocytic cup formation in murine macrophages (RAW 264.7) deficient in INPP5E 21. Furthermore, the expression of a constitutively active form of RAB5B in these cells rescues PI(3)P accumulation 21. Therefore, INPP5E plays a crucial role in RAB5 activation by functionally interacting with RAB20 in phagosomes, leading to increased PI(3)P levels and delayed phagosome acidification 21.

### 5.3. Role of INPP5E in autophagy

Autophagy is a natural cellular process in which cells break down and recycle their components. It plays a crucial role in maintaining cellular health and homeostasis by removing cellular debris, recycling damaged components, and providing energy and building blocks for cellular renewal and repair 19, 20. Autophagy is also involved in various physiological processes, such as development, growth, immunity, and adaptation to nutrient availability and stress conditions 19, 20. During autophagy, autophagosomes engulf damaged organelles, misfolded proteins, and other cellular waste 20; they then fuse with lysosomes to break down the engulfed materials into their basic building blocks 20. INPP5E plays a crucial role in the fusion of autophagosomes and lysosomes in neuronal cells 20. Lysosomes require actin filaments on their surface for fusion with autophagosomes, and activated cortactin stabilizes these filaments 20. INPP5E catalyzes PI(3,5)P2—a phosphatase substrate that counteracts cortactin-mediated actin filament stabilization—to PI(3)P in lysosomes, decreasing PI(3,5)P2 levels and promoting cortactin activation 20. Cortactin binds to actin filaments, stabilizing them on lysosomes and ensuring membrane anchoring and autophagosome-lysosome fusion 20. Consequently, *INPP5E* knockdown impairs autophagosome-lysosome fusion, markedly inhibiting autophagy 19, 20.

### 5.4. Role of INPP5E in olfactory sensory neurons

The lipid composition of the primary ciliary membrane is a crucial factor regulating cilium formation, maintenance, and function 34. In mammals, odors are recognized through the conversion of chemicals into neural signal cues by olfactory sensory neurons 34. When odor molecules activate olfactory cilia, PI(3,4,5)P3 levels are decreased by INPP5E, which stops Ca^2+^ influx and redistributes PI(3,5)P2 34. This redistribution of PI(3,5)P2 enhances the sensitivity of the neurons to odorants by increasing the exposure of receptors to the extracellular environment 34. Conditional knockout mice with *Inpp5e* deletion in olfactory sensory neurons (OSNs) exhibit 34 impaired odor adaptation and reduced recovery from the short-lived excitation. Deletion of *Inpp5e* in mouse OSNs decreased cilia numbers and length, increased PI(3,4,5)P3 levels, altered PI(3,5)P2 distribution, and resulted in AKT signaling pathway activation and tremendous intraciliary Ca^2+^ elevation 34. These findings demonstrate that INPP5E is involved in the modulation of odorant receptor trafficking and signaling via the regulation of PI(3,5)P2 levels to trigger the redistribution of odorant receptors within the plasma membrane of OSNs 34.

### 5.5. Role of INPP5E in mitosis

The life cycle of a primary cilium, which begins at the quiescence stage and ends before mitosis, has been extensively studied 23, 45, 46. Sierra Potchanant *et al.* 23 reported that INPP5E is essential for maintaining cellular homeostasis and development via cell division regulation 23. INPP5E silencing or knockout in murine/human cells weakens spindle aggregation checkpoint, centrosome formation, spindle function, and chromosomal integrity. Additionally, INPP5E expression is cell cycle-dependent, heightening during mitotic entry 23. INPP5E protein localizes to chromosomes, centrosomes and kinetochores during early mitosis and transports to the midzone spindle at the mitotic exit 23. Upon nuclear envelope breakdown, INPP5E diffuses from the nucleus through the cell, with a portion accumulating around chromosomes; its abundance increases during the prophase 23. INPP5E translocates to the nucleus during telophase after nuclear envelope reformation 23. Moreover, INPP5E prevents aneuploidy. *INPP5E* knockdown causes chromosomal instability in primary human fibroblasts, a hallmark of carcinogenesis or cellular dysfunction. However, INPP5E transcription is reportedly upregulated in cervical cancers, uterine leiomyomas, and lymphomas and downregulated in gastric carcinomas and metastatic adenocarcinomas 23. Further studies are needed to better understand the true impact of INPP5E's role in the chromosome 23. Nevertheless, these findings highlight the crucial role of INPP5E in mitosis and aneuploidy prevention.

INPP5E also regulates the cilia life cycle and cell cycle through cilia decapitation to induce cell mitogenic signaling 45. Deletion or mutation of *INPP5E* causes PI(4,5)P2 accumulation at the distal cilia, leading to cilia decapitation; that is, the excision of cilia tips from cilia 45. Moreover, Aurora kinase A (AURKA) functions downstream of INPP5E and HDAC6 in complementary pathways to regulate cilia decapitation. AURKA phosphorylates INPP5E to modulate its 5-phosphatase activity and governs the re-localization of INPP5E, suggesting that AURKA may have a broader role in coordinating ciliary dynamics and signaling 45.

Beside regulating cell cycle, INPP5E also regulates extracellular vesicles, including exosomes and micro-vesicles, that participate in intercellular communication within a single organism or between different organisms, species, and kingdoms 46. Distal cilia tips are shed or “decapitated” when there is growth stimulation or high levels of ciliary PI(4,5)P2. During growth induction, PI(4,5)P2 accumulates at the distal ends of cilia and displaces the ciliary protein INPP5E 45, 46. This displacement triggers actin (F-actin) polymerization, which enters the primary cilia, leading to decapitation 45, 46. Although cilia disassembly is traditionally thought to occur through resorption, the acute loss of IFT-B protein components (ARL13B, IFT88, and IFT81) due to cilia decapitation precedes resorption 45. In summary, the exit of INPP5E from the cilium leads to F-actin accumulation at the sites of ciliary decapitation 45, 46. Concurrently, the shedding of extracellular vesicles from decapacitated cilia precedes cilia disassembly and influences the cell cycle 46.

## 6. INPP5E regulation in signaling pathways and other mechanistic analyses

**Figure 4:**
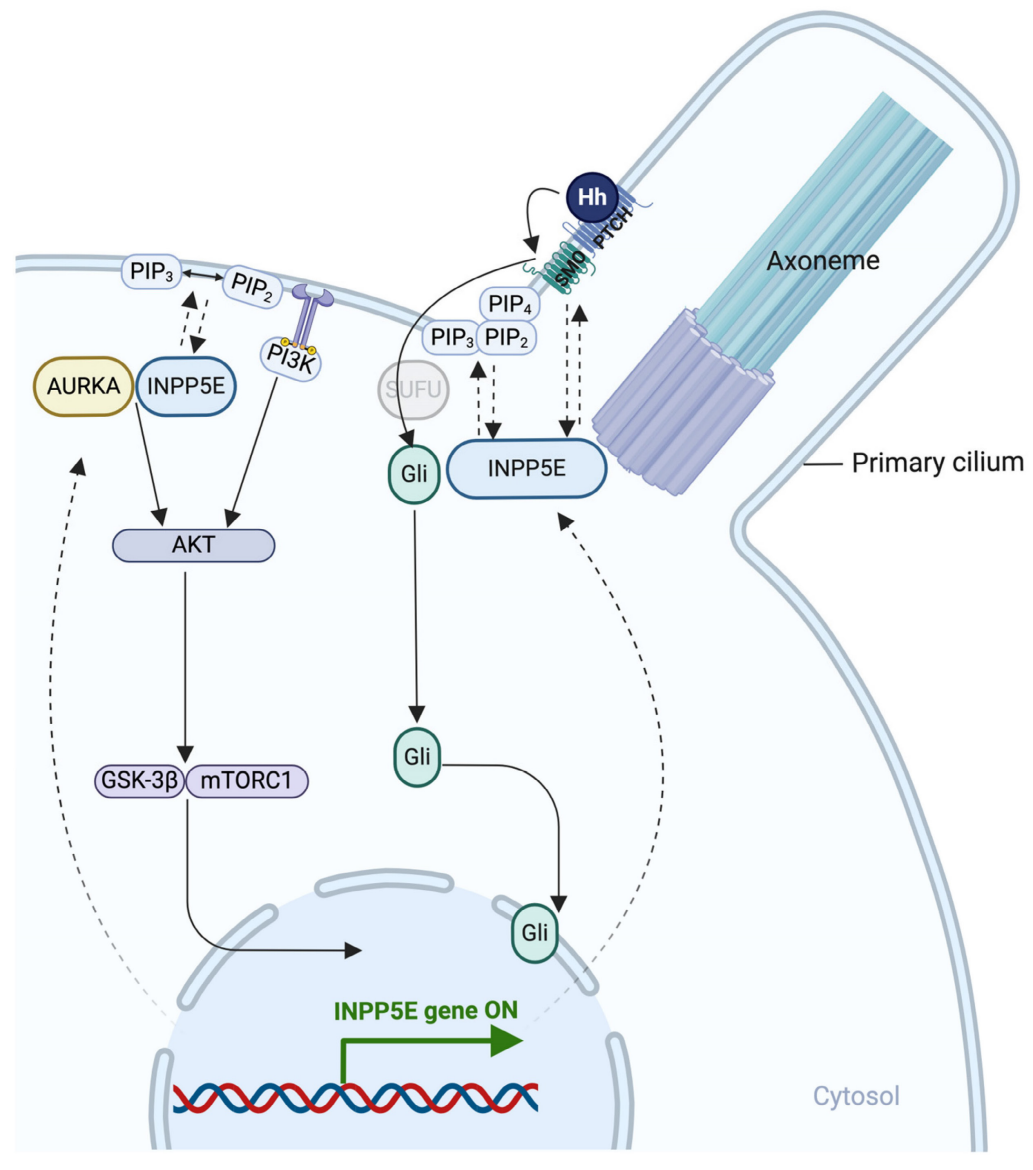
** The main pathways involved in INPP5E activation.** Shh signaling pathway. In the unstimulated state, patched (PTCH) localizes in the cilium membrane, inhibiting SMO entry. GLI family zinc finger (GLI) proteins are suppressed by SuFu at the ciliary tip. Upon SHH binding to PTCH, SMO repression is released, allowing its entry while PTCH remains in the cilium. SMO then represses SuFu, enabling GLI activation. Activated GLI (GLIA) translocates to the nucleus, driving target gene expression. During Hh pathway activation, phosphatidylinositol-4,5-bisphosphate 5-phosphatase E (INPP5E) helps regulate SMO and GLI2/GLI1 translocation. Moreover, INPP5E regulates GLI3 processing, possibly due to increased PI(3,4,5)P3. PIP2 and PIP3 activate the SHH signaling pathway to control downstream target gene expression. INPP5E may also influence GLI3 processing by impacting the transition zone (TZ). TKR signaling pathway: INPP5E can mediate tyrosine kinase receptor (TKR) pathway activation by regulating Aurora kinase A (AURKA) via phosphatidylinositol 4,5-bisphosphate (PIP2) control and PIP3 conversion, activating signaling cascades, including protein kinase B (AKT), glycogen synthase kinase-3 (GSK-3)β, and mammalian target of rapamycin (mTOR)C1. Subsequently, *INPP5E* expression is upregulated. *Created in BioRender. Hakeem, A. (2024) BioRender.com/y60b182*.

The interactions, associations, and regulatory roles of INPP5E make it essential for maintaining cellular functions and processes, particularly in ciliated cells. In addition to the Hh pathway, INPP5E functions through the GSK-3β, Wingless-related integration site, PI3K, and nuclear factor kappa B (NF-κB) pathways, with particular importance in the PdtIns(3,4,5)P3/PI3K/AKT and Hh signaling pathways (Fig. 4). The primary function of INPP5E in cilia is to produce a PI(4)P-rich membrane environment by converting PdtIns(4,5)P2 to PI(4)P, which is crucial for controlling the localization of receptors involved in the Hh signaling pathway. Although further studies are needed to elucidate the mechanisms underlying the roles of INPP5E and develop restorative interventions for ciliopathies, some important findings have been published, as presented herein.

### 6.1. PI(3,4,5)P3/PI3K/AKT pathway

INPP5E regulates PI(3,4,5)P3 hydrolysis, which is required for appropriate AKT activation 24, 37. At the primary cilia, the hydrolysis of PI(3,4,5)P3 by INPP5E changes the different phosphoinositide levels in species and facilitates the functional interaction between INPP5E and AURKA, activating AURKA through auto-phosphorylation 23, 37. When this pathway is activated, INPP5E hydrolysis of PI(3,4,5)P3 facilitates the binding of AURKA to INPP5E and its phosphorylation, thus increasing INPP5E's phosphatase activity 23, 44. This process subsequently reduces AURKA transcription downstream of AKT 23, 44. AURKA phosphorylates INPP5E, enhancing its activity and suppressing the PI3K/AKT pathway 23, 37. Ciliary INPP5E is required for PI(4,5)P2 hydrolysis in cilia and subsequent activation of the Shh pathway 24, 30-33, 35. In the absence of INPP5E, upregulated PI(3,4,5)P3 levels increase AKT phosphorylation, suggesting that INPP5E also regulates PI(3,4,5)P3 outside cilia 23, 24, 37.

### 6.2. Hh signaling pathway

The Hh pathway is a primary cellular formation and development pathway 30, 35. In the absence of Hh, patched (PTCH) is localized on the cilia and cell membrane, preventing high expression of SMO, a G protein-coupled receptor, trafficking to cilia. Hh binds to PTCH to release the inhibition in SMO expression and translocation to cilia. Hh activates SMO and converts the signal cascade to INPP5E activation^30^, ^35^. SMO accumulation is increased in wild-type mice, promoting *Inpp5e* activation, i.e., positive regulation 30, 35. In contrast, reduced *Inpp5e* expression in neural and NIH3T3 cells in mice causes primary cilia malformation and neural maldevelopment 35. Furthermore, *Inpp5e* deletion suppresses Hh signaling in mouse embryos despite an average number of cilia and low SMO levels 35. This low SMO level in the cilia is due to TZ diffusion failure as a gatekeeper 30, 35. SMO translocates into cilia to activate Hh signaling; in contrast, reduced ciliary localization of SMO reduces its ability to activate Hh signaling 30, 35. At the TZ, INPP5E is important in regulating PI(4,5)P2 and PI(3,4,5)P3, which influence SMO accumulation and Hh signal activation 30. *INPP5E* deletion negatively affects the accumulation of SMO, GLI family zinc finger 2 (GLI2), IFT, and other proteins at the ciliary base in human neural cells 40. However, as a compensatory response, SHH signaling accumulates at the site of *INPP5E* deletion, leading to the formation of more neurons and progenitors 40. Moreover, the development of certain diseases may be attributed to abnormalities in the SHH signaling pathway, potentially due to ciliary dysfunction 40. Further research is needed to determine the effects of INPP5E dysfunction, such as its impact on inositol phosphate and other signaling pathways.

Additionally, it is vital to understand how the various cellular defects associated with the loss of *INPP5E* function are related to human phenotypes 47. In cells cultured from mice harboring an inactivated *Inpp5e* allele, MEFs exhibit no *Shh* transcriptional response, whereas neural cells exhibit an enhanced *Shh* transcriptional response 33. *Inpp5e* weakens SHH signaling in developing mouse neural cells and enhances it in MEF cells 33. SMO, PTCH, and SuFu keeps the pathway in an “off” state while the ligand is absent; therefore, their loss completes the pathway activation besides +GliA/-GliR production 33.

Further studies should address how Inpp5e helps Hh ligand binds to its receptor PTCH. We think that PTCH saturation on the ciliary membrane, causing its removal from the cilium, permitting the GPCR SMO entry 48. The current understanding is that PTCH remains localized on the cilia and cell membrane without SHH stimulation. In contrast, with SHH, PTCH disassociates from the cilia, and SMO translocates into the cilia.

Moreover, the need to understand the ciliary expression of SUFU and GLI in relation to Inpp5e protein during Hh signaling is essential. We think that the activation of Hh signaling leads GLI and SUFU entering the cilium and gathering at the ciliary tip. In activation, SHH binds to PTCH to release the inhibition in SMO; then, SMO, SUFU, GLI proteins accumulate at the ciliary tip 49. There, the GLI proteins phosphorylates to transcriptional activators that move to the nucleus and incite the target genes transcription of SHH pathway 49.

The deletion 27, 30 (germline deletion, precursors granule cell neurons deletion) of INPP5E in mice reduces the ciliary localization of GLI2/GLI1 and SMO during Hh pathway activation. Moreover, in this pathway, the Hh ligand binds to its receptor PTCH in the ciliary membrane 18 to trigger the removal of PTCH from the cilium, enabling SMO, a GPCR, to translocate into cilia. This action results in the cleavage and initiation of GLI transcription factors, which then move to the nucleus and initiate the target genes transcription 18. *Inpp5e* is also involved in controlling direct and indirect neurogenesis through GLI3 processing 50. In *Inpp5e* mutant embryos, the GLI3R level and GLI3R/GLI3FL ratio decrease, increasing the prevalence of direct neurogenesis 50. The loss of INPP5E results in structural abnormalities in cilia, possibly due to increased PI(3,4,5)P3. INPP5E may also influence GLI3 processing by impacting the TZ. These findings suggest that INPP5E is crucial in regulating cilia formation and function and GLI3R formation 50.

Taken together, *Inpp5e* regulation of the Hh response involves a more complicated mechanism than previously appreciated, and it differs from one cell type to another 24, 30-33, 35, 50.

### 6.3. Interactions between *INPP5E* and other proteins

INPP5E exhibits complex action mechanisms in the cilia and interacts with many proteins (ARL13B, ATG16L1, CEP164, IFT20, PDE6D, retinitis pigmentosa GTPase regulator (RPGR), and TULP3) to achieve ciliary homeostasis 38, 51, 52. Dynamic equilibrium between these proteins and INPP5E is essential to maintain normal function within the cilia and promote cell survival 38, 51, 52. Accordingly, the interactions between INPP5E and different proteins and the effects of these interactions on cell function have garnered substantial research interest 50, 51.

PDE6δ is important in ciliary sorting and localization mechanisms. PDE6δ sorts farnesylated INPP5E into cilia through high-affinity binding and release by the ADP-ribosylation factor (ARF)-like protein ARL3·GTP1 38. PDE6δ-free INPP5E can be specifically retained in the cilia 50. INPP5E is transported to the ciliary base upon binding to PDE6δ 51. After diffusing into the cilium, INPP5E is released from PDE6δ by ARL3·GTP, and the farnesyl moiety connects it to the ciliary membrane 51. INPP5E is then carried by the IFT system and retained inside the cilium 38, 50. ATG16L1 partially affects the primary ciliary phosphoinositide equilibrium 38, 51. However, it directly regulates the distribution of ciliary phosphoinositides and INPP5E trafficking, which is associated with primary cilia membrane regulation 17. Humbert *et al.* 53 investigated an ARL13B/PDE6D/CEP164 complex to track the ciliary location of INPP5E. ARL13B and PDE6D are ciliary proteins that connect to INPP5E, directing them toward the target area inside cilia 54.

Other specific carrier proteins also facilitate INPP5E transportation to cilia 38, 54. ARL13B—a small GTPase—also contributes to the localization of INPP5E, although the precise mechanism is not fully understood 38, 54. Once inside the cilia, the inner ciliary transport of INPP5E is regulated solely by the IFT system, independent of PDE6δ activity and INPP5E farnesylation 38. Therefore, INPP5E depends on these carrier proteins and transport systems for its localization and movement within the cilia 38, 54. In particular, TULP3 plays a crucial role in the localization of phosphoinositide phosphatase INPP5E in the cilia 54. It interacts with the intraflagellar IFT-A, which is responsible for the ciliary localization of transmembrane proteins 54. TULP3 is an adapter for transporting various integral membrane cargo to the cilia 54. The Tubby domain of TULP3 binds to PI(4,5)P2—a phosphoinositide—essential for retrograde ciliary protein trafficking mediated by IFT-A and TULP3 54. In the absence of ciliary INPP5E, particularly under PI(4,5)P2-rich conditions, this trafficking is impaired 38, 54. Furthermore, TULP3 regulates the association of INPP5E with other proteins, such as ARL13B. This complex interplay ensures the correct localization of INPP5E in the cilia. Hence, TULP3 acts as a carrier of INPP5E to the cilia, playing a critical role in its ciliary localization and function 54.

### 6.4. Mechanisms of INPP5E ciliary targeting and localization

INPP5E functions as a basic controller and effector in many cells, especially those with cilia 36, 37, 55. On ciliary cells, such as murine embryonic fibroblasts, human embryonic kidney cells, and retinal pigment epithelial cells, INPP5E targets an intricate interaction of proteins, such as ARL13B, PDE6D, and CEP164, ensuring its localization to the targeted site 36, 37, 55. Deletion of INPP5E causes cilia loss and alters the total level of AKT, upregulating AURKA transcription, a centrosomal kinase that regulates mitosis and ciliary disassembly, resulting in its accumulation in the remaining cilia 37. In response to physiological stimuli, an increased pool of AURKA at this site drives an abnormal cilia disassembly phenotype 37. Meanwhile, AURKA inhibition may rescue the defects caused by INPP5E loss 37. The interaction between INPP5E and AURKA at the primary cilia may occur as follows 37. PI(3,4,5)P3 hydrolysis by INPP5E alters the levels of different phosphoinositide species, mediating the functional interaction between INPP5E and AURKA and activating AURKA via auto-phosphorylation. Activated AURKA attaches to and phosphorylates INPP5E, rising its phosphatase levels and decreasing AURKA transcription downstream of AKT 37. In the case of INPP5E deletion, elevated AKT signaling increases AURKA transcription, causing in a general AURKA buildup at the cilia. Responding to physiological stimuli, the heightened AURKA accumulation may drive the abnormal cilia disassembly phenotype seen in cells depriving INPP5E 44. Moreover, Fujisawa *et al.* 36 highlighted the association between ARL3 and ARL13B GTPases and the different forms of INPP5E 36. Their findings underscore the multifaceted nature of the INPP5E restriction cycle 36. The association of INPP5E with ARL13B is fundamental in maintaining ciliogenesis and supporting ciliary homeostasis 36. RPGR isoforms play a crucial role in regulating binding partners in the primary cilia, potentially affecting the functional properties of INPP5E and RPGR-interacting protein 1-like (RPGRIP1) 55. RPGR may act as a molecular link that fine-tunes the functions of these binding partners in the primary cilium 55.

## 7. *INPP5E* mutations in human diseases

A ciliopathy is a genetic disorder that affects the cilia structures (basal bodies) and/or function 31, 56-66. These disorders are characterized primarily by several unrelated genes, such as *INPP5E, NPHP1, AHI1, CEP290, CSPP1, KIF7, TCTN1, OFD1, and ARL13B,* that affect the ciliary structure or function 31, 56-66. *INPP5E* mutations are predominantly homozygous; the following mutations have been reported in the literature: R378C, G286R, V303M, R345S, T426N, R512W, R435Q, W474R, R515W, Y534D, R563H, K580E, R585C, Y588C, R621, C641R, Q309X, Y543X, and Q627X 56. Diseases linked to *INPP5E* mutations include Joubert syndrome, Leber congenital amaurosis, and MORM syndrome. However, many other diseases, including PKD, SHH medulloblastoma, cystic renal dysplasia, hepatic fibrosis, colorectal carcinoma, IRD, monogenic obesity syndrome, and Senior-Loken syndrome, are associated with *INPP5E* mutations 31, 56-66.

### 7.1. Joubert syndrome

Joubert syndrome is a rare, autosomal recessive, X-linked disorder of neurological impairment characterized by mid- and hindbrain malformation, hypo-dysplastic cerebellar vermis, and neuro-dialogical (structural) or molar tooth sign 16, 67. This disorder is accompanied by dysregulated breathing patterns, ataxia, and delayed development 16, 67. Typical neurological signs in infants include dysregulated breathing patterns, ataxia, hypotonia, apnea, oculomotor dysfunction/ocular anomalies (reported in all cases with the *INPP5E* mutation), delayed psychomotor function, and multi-organ anomalies involving the digits, eyes, liver, and kidneys 16, 67. Joubert syndrome is characterized by clinical and genetic heterogeneity and is associated with mutations in the following genes: *INPP5E*, *NPHP1*,* AHI1*,* CEP290*,* CSPP1*,* TCTN3*,* MKS11*,* MKS10*,* MKS9*,* TCTN2*,* NPHP3*,* CC2D2A*,* RPGRIP1L*,* MKS4*,* TMEM67*,* TMEM216*,* MKS1*,* CEP120*,* C2CD3*,* B9D2*,* B9D1*,* MKS1*,* PDE6D*,* IFT172*,* TCTN3*,* C5ORF42*,* TMEM231*,* TMEM138*,* CEP41*,* TMEM237*,* KIF7*,* TCTN1*,* OFD1*, and *ARL13B* 68.

Mutations in *INPP5E* may lead to the formation of an unstable protein, resulting in reduced levels of INPP5E in the fibroblasts and impaired ciliary development 33. Biochemical data from patients with Joubert syndrome has shown a missense mutation in *INPP5E*, which is associated with a reduced number of cilia and shorter ciliary length, indicating that INPP5E is essential for cilia formation and function 33. INPP5E coordinates with the centrosomal enzyme Ptdlns(4)P 5′-kinase or PIPKly to initiate cilia formation 33. *INPP5E* deletion suppresses cilia formation, resulting in TULP3 and G protein-coupled receptor 161 accumulation, which represses the SHH signaling pathway and downregulates phosphoinositide production 33. ARL13B, CEP164, and PDE6D are involved in the ciliary localization of INPP5E; mutations in *INPP5E* lead to the pathological alterations underlying Joubert syndrome, resulting from differences in cilia length and localization patterns 47.

Mutations in *INPP5E* are associated with a limited genotype-phenotype correlation but pronounced phenotypic variation in Joubert syndrome 69. For example, because INPP5E is necessary for ciliary signaling pathway regulation, mutations in *INPP5E* result in cilium dysfunction and dysregulated brain development, specifically of the mid- and hindbrain, which are crucial for body coordination, ocular and renal function (preventing cystic kidneys), breathing pattern maintenance, and intellectual ability 69. An unusual homozygous mutation in *INPP5E,* c.1303C>T, leads to the replacement of arginine by tryptophan at residue 435 (p.Arg435Trp), affecting protein functionality and altering brain function 68, 70.

*INPP5E* regulates many of proteins localized in the TZ, which is located in the proximal region of the cilia 66,68. This zone begins at the distal end of the ciliary basal and links to the plasma membrane and axoneme which allows the movement of proteins “in-and-out” of the cilium 71.

Joubert syndrome and related anomalies represent a group of genetically heterogeneous, cilium-related pathologies characterized by a cerebellar deformity known as the molar tooth sign on brain MRI 72. Furthermore, *INPP5E* mutations are related to dysfunctional cilia formation or ciliopathy, dysregulated cilia signaling pathways, structural abnormalities in the brain, heterogeneity in genes leading to Joubert syndrome onset, and the related clinical signs and symptoms shown by Joubert syndrome patients 72. Gene profiling of patients with Joubert syndrome has revealed interactions between different genes with *INPP5E* in modulating ciliary function; mutations in *INPP5E* downregulate phosphoinositol metabolism 73. Exome and next-generation sequencing have been used to analyze *INPP5E* mutations related to Joubert syndrome and associated syndromes 73. This has led to the development of a novel diagnostic technique based on the identified heterozygous *INPP5E* variations 73, emphasizing the importance of molecular and genetic testing for achieving a precise diagnosis of this complex and rare neurociliopathy.

### 7.2. MORM syndrome

MORM syndrome is a rare condition in men that is characterized by obesity, micropenis, intellectual disability, and retinal dystrophy 56, 74. The gene affected by this autosomal recessive disorder is located on chromosome 9q34.3 56, 74. As *INPP5E* is located at this same chromosomal position, the condition has been linked to *INPP5E* 56, 74. Although the exact mechanistic role of *INPP5E* in the disease has not been defined, it has been hypothesized that it may involve defective primary cilia 56, 74.

### 7.3. Other ciliopathies

Disorders characterized by primary ciliary dysfunction caused by genetic mutations are termed ciliopathies 75. Ciliopathies can include brain anomalies, kidney cysts, retinal degeneration, obesity, polydactyly, and infertility 50, 76, 77. Control of ciliary phosphoinositide metabolism is crucial for mammalian development and is essential for maintaining tissue integrity in adulthood, as lack of control due to *INPP5E* mutations can lead to ciliopathies 75. Mutations in *INPP5E* affect various cell types that rely on ciliary signaling, such as neural stem cells 50, ciliated epithelial cells 15, photoreceptor cells 76, and kidney cells 77. In the primary cilia, INPP5E modifies the functions of the PI3K/AKT, Hh, and GSK-3β signaling pathways, markedly affecting cell development, viability, and specialization.

#### 7.3.1. Neural maldevelopment

The role of INPP5E in ciliopathy has been investigated using various animal and human models. In mice 50, *Inpp5e* deletion leads to increased cortical generation of basal neuronal progenitors. INPP5E influences the balance between indirect and direct neurogenesis, and its absence causes cortical thinning and reduced brain size 50. Furthermore, INPP5E regulates the ciliary localization of GLI3, a transcription factor that mediates SHH signaling 50. This process is necessary for the morphogenesis and patterning of different tissues. Hasenpusch-Theil *et al.* 50 highlighted the short-term function of INPP5E and its association with GLI3 in regulating direct and indirect neurogenesis during cortical development. Hoang *et al.* 44 reported an alternative 5′ leader of *Inpp5e* mRNA that enhances its translation under stress conditions and demonstrated its impact on cellular resistance to infection by oncolytic viruses. Meanwhile, Khan *et al.* 77 identified two new *INPP5E* variants in families with ciliopathic illnesses in Pakistan and expanded the genetic and clinical ranges of Bardet-Biedl syndrome and Joubert syndrome. Rao *et al.* 76 studied the relationship among RPGR, PDE6, and INPP5E, as well as their roles in the homeostasis and composition of ciliary proteins in neural maldevelopment. They detected RPGR in cilia; its localization in the cilia is vital for its functions. PDE6δ interacts with RPGR, which is essential for RPGR's localization in cilia. INPP5E interacts with RPGR and PDE6δ, and the transportation of INPP5E to cilia depends on RPGR's ciliary localization and interaction with PDE6δ. Moreover, Yinsheng *et al.* 78 studied the role of transmembrane protein 67 in gating ARL13B and INPP5E proteins across the primary cilia membranes. ARL13B interacts with and helps target INPP5E to cilia. Disruption of this interaction by ARL13B missense mutations leads to impaired neural development. These proteins form a functional network crucial for ciliary function and neural development 50, 76-78.

#### 7.3.2. Polycystic kidney disease (PKD)

PKD causes progressive dilation of renal tubules and kidney enlargement, eventually leading to renal failure 57. The symptoms include high blood pressure, side or back pain, and a swollen abdomen 57. It is linked primarily to *PKD1* and *PKD2;* however, germline *Inpp5e* deletion may also be linked to PKD 22, 58. The loss of INPP5E in the kidney epithelium leads to aggressive PKD development. Meanwhile, AKT hyperactivation activates the mammalian target of rapamycin (mTOR) complex 1 signaling in renal epithelial cells 57, 58. Additionally, INPP5E is a key inhibitor of PI3K/AKT and acts downstream in mTORC1 signaling in the kidney epithelium *in vivo* 57, 58. Thus, dual PI3K/mTORC1 inhibition may be effective for identifying kidney-related pathologies and therapies 22. Treatment includes tolvaptan, an agonist for cAMP generation, and mTOR inhibitors 57.

#### 7.3.3. SHH medulloblastoma

Medulloblastoma is a highly malignant brain tumor typically affecting children 59. It originates from embryonal cells in the cerebellum and can arise from various stem cell or progenitor cell populations in early development 59. Although it can occur during infancy or adulthood, the typical age of diagnosis is 6-8 years 59. The SHH medulloblastoma subgroup is well-defined genetically, with most patients carrying somatic or germline mutations and copy-number alterations in critical genes of the SHH signaling pathway 27, 59. The best survival rates have been achieved through maximal surgical resection and adjuvant chemotherapy 59. Meanwhile, *INPP5E* is a crucial regulator of primary ciliary dynamics that promotes the progression of SHH medulloblastoma 27. *INPP5E* expression was downregulated in a subset of patients with SHH medulloblastomas and correlated with enhanced overall survival 27. INPP5E confines to the primary cilia, regulating a compartmentalized PI3K/AKT/GSK-3β signaling axis in medulloblastoma and maintain the primary cilia 27. The loss of *INPP5E* causes elevated PI(3,4,5)P3, AKT, and GSK-3β levels in cilia 27. A decreased number of ciliated cells leads to reduced SHH signaling and a subsequent switch from a proliferative to a differentiated phenotype, resulting in slower tumor progression 27.

#### 7.3.4. Cystic renal dysplasia and hepatic fibrosis

Neonatal mortality in Norwich terriers is caused by renal cystic dysplasia and congenital hepatic fibrosis 31. *INPP5E* c.1572+5G>A is responsible for hepatorenal fibrocystic disorder in Norwich terriers 31. The affected dogs and carriers are part of an isolated Finnish family with a common ancestor tracing back 15 generations 31. This genetically defined syndromic ciliopathy leads to neonatal mortality in Norwich terriers 31.

#### 7.3.5. Inherited retinal diseases (IRDs)

IRDs are a group of disorders that affect the eyes and other parts of the body, including the central nervous system, ear, skeleton, kidney, and cardiovascular system 61, 62. In some cases, they cause inborn errors of metabolism and ciliopathies 61, 62. These diseases are caused by various genetic mutations 61, 62. Currently, 200 genes, including *INPP5E,* have been identified as associated with IRDs 61, 62. Many of these genes are rare and inherited in a recessive manner 61. Defects in *INPP5E* can lead to early-onset, non-syndromic IRD 61, 62. Sangermano *et al.* reported 16 non-syndromic patients with IRD from ten families and two mildly syndromic Joubert syndrome (JBTS) cases, all with rare *INPP5E* gene variants 62. They identified 14 *INPP5E* variants, of which 12 were novel, mainly missense changes in conserved amino acid residues within the phosphatase catalytic domain, suggesting a broader genetic diversity in INPP5E-associated diseases 62. Although INPP5E variants are linked to syndromic and non-syndromic cases, no clear correlation between specific genetic changes and disease severity was observed, indicating that additional genetic factors may modify the clinical outcomes 62. Meanwhile, in zebrafish 4, *inpp5e* knockdown causes defects in membrane left-right asymmetry, eye development, and pronephric duct formation. In humans, *INPP5E* mutations impair phosphatase activity and ciliary stability, disrupting ciliary function and development 61, 62. These findings demonstrate the broad phenotypic spectrum of *INPP5E*-related diseases 61, 62.

#### 7.3.6. Leber congenital amaurosis (LCA)

LCA is a severe IRD and a genetic disorder causing vision loss in children 63. Over 38 genes have been identified as the cause of LCA, including *INPP5E* and *RPE65*, resulting in a wide range of clinical symptoms 63. Clinical assessment, fundoscopic images, optical coherence tomography examination, and electroretinogram tests can help establish the correct diagnosis 63. Moreover, gene therapy holds promise for LCA, with various clinical trials focusing on other LCA-related genes to treat the condition 63.

#### 7.3.7. Senior-Loken syndrome

A recent study presented a case of Senior-Loken syndrome attributed to the deletion of a critical TZ protein that ultimately leads to renal failure and retinal degeneration 66. This report offered critical insights into primary ciliary morphological aberrations in a renal biopsy, which resulted in impaired function 66. Moreover, the study highlighted altered ciliary localization of *INPP5E* in a patient with nephrocystin 1 (*NPHP1*) deletion 66. Abnormal expression of *NPHP4* was also detected in human *NPHP1-*deficient tissues, underscoring the crucial role of *NPHP1* in TZ formation, where *NPHP4* functions normally 66. This helps regulate *INPP5E* transition and its ability to influence cilia stability, providing crucial insights into the pathophysiology of Senior-Loken syndrome 66.

#### 7.3.8. Monogenic obesity syndrome

A recent case study reported that a Caucasian girl diagnosed with monogenic obesity syndrome was attributed to a novel *INPP5E* truncating variant 64. The study identified a cone-rod-type progressive retinal dystrophy that emerged during the first decade of the patient's life 64. In addition, severe obesity was observed within the first year of life, leading to the early onset of prediabetes, arterial hypertension and, dyslipidemia 64. Notably, a delay in acquiring language skills was observed 64. These findings are important for prognosis and informed treatment decision-making 64.

Another study demonstrated the involvement of INPP5E in insulin signal transduction in muscle and adipose tissue. Reduced INPP5E levels enhance insulin signaling by regulating PI3K/AKT and FOXO1 signal transduction 65. As a glimpse of the potential application of *INPP5E* in therapy, Bertelli *et al.* 65 found that reducing *Inpp5e* expression through the PI3K pathway in mice and rats resulted in reduced weight gain and improved insulin production to manage blood sugar in obese animals. This finding opens numerous possibilities for treating obesity and diabetes.

#### 7.3.9. Colorectal carcinoma (CRC)

CRC is a common cancer with risk factors including age, chronic disease history, lifestyle, and gut microbiota 26, 60. CRC is triggered by mutations in oncogenes, tumor suppressor genes, and mechanistic error in DNA repair genes 26, 60. In CRC, chromosomal changes, common mutations, and translocations have been reported to influence major pathways (such as WNT and PI3K), and mutations, such as PIK3CA, can be used to predict prognosis 60. *INPP5E* is linked to CRC through miR-598, which is frequently upregulated in CRC. miR-598 overexpression promotes cell proliferation and cell cycle progression in CRC by silencing *INPP5E* 26. This provides a better understanding of the molecular pathways underlying CRC pathogenesis 26.

## 8. Conclusion

In this review, we have summarized the reported cellular pathogenesis pathways associated with *INPP5E*. However, the specific mechanisms for different cell types and tissues warrant further investigation. Moreover, whether INPP5E can move independently to the cilia and the carrier molecules responsible for its transport require evaluation. This may provide further insights into the levels of PI(4,5)P2 and INPP5E proteins in cilia, ultimately characterizing the specific mechanism(s) underlying cilia formation.

Although the role of inositol deficiency in ciliogenesis is known, the contributions of INPP5E-specific mechanisms remain to be elucidated. To this end, the specific mechanisms under deficient conditions among different cells must be defined. Moreover, *INPP5E* is essential for ciliogenesis initiation and coordination. However, the precise mechanism(s) and role(s) of INPP5E in cilia formation are highly complex, particularly among cell types and tissues. A better understanding of the molecular and cellular mechanisms of INPP5E in regulating development and diseases could inform the development of novel therapeutic approaches.

Furthermore, promising results have demonstrated the potential role of INPP5E in lowering weight and improving insulin production to manage blood sugar in obese animals 64. Nonetheless, further studies are required to gain a complete understanding of the therapeutic role of INPP5E in animal and human models using various cells, tissues, and treatment conditions. Finally, proteomic studies and pathway analysis may help elucidate the regulatory cross-talk between INPP5E and other proteins involved in cellular signaling pathways, clarifying how these interactions influence ciliary dynamics.

## Abbreviations

AKT: protein kinase B
ARL: ADP-ribosylation factor-like GTPase
AURKA: Aurora kinase A
CC: connecting cilium
CEP164: centrosomal protein 164
GLI3: GLI family zinc finger 3
GSK-3β: glycogen synthase kinase 3β
Hh: Hedgehog
IFT: intraflagellar transport
INPP5: inositol polyphosphate 5-phosphatase
IRD: inherited retinal degeneration
MEF: mouse embryonic fibroblast
MORM: mental retardation, truncal obesity, retinal dystrophy, and micropenis
mTORC1: mammalian target of rapamycin complex 1
NPHP1: nephrocystin 1
OS: outer segment
P10: photoreceptor 10
PDE: phosphodiesterase
PI3K: phosphoinositide 3-kinase
PIPP: proline-rich inositol polyphosphate 5-phosphatase
PIPKI: phosphatidylinositol-4-phosphate 5-kinase type I
PIPKly: Ptdlns(4)P 5′-kinase
PKD: polycystic kidney disorder
PI(3,4)P2: phosphatidylinositol 3,4-bisphosphate
PI(3,5)P2: phosphatidylinositol 3,5-bisphosphate
PI(3,4,5)P3: phosphatidylinositol (3,4,5)-trisphosphate
PI(4)P: phosphatidylinositol 4-phosphate
PI(4,5)P2: phosphatidylinositol 4,5-bisphosphate
RPGR: retinitis pigmentosa GTPase regulator
Shh: Sonic Hedgehog
SMO: smoothened
TTBK2: tau tubulin kinase 2
TULP3: tubby-like protein 3
TZ: transition zone
